# Inter-Rater Reliability of Collateral Status Assessment Based on CT Angiography: A Retrospective Study of Middle Cerebral Artery Ischaemic Stroke

**DOI:** 10.3390/jcm12175470

**Published:** 2023-08-23

**Authors:** Daria D. Dolotova, Evgenia R. Blagosklonova, Rustam Sh. Muslimov, Ganipa R. Ramazanov, Tatyana A. Zagryazkina, Valentin N. Stepanov, Andrey V. Gavrilov

**Affiliations:** 1 Department of Bioinformatics, Department of Pediatric Surgery, Pirogov Russian National Research Medical University, Russian Ministry of Health, 117997 Moscow, Russia; 2 Research Department, Gammamed-Soft, Ltd., 127473 Moscow, Russia; 3 Department of Radiology, Scientific Department of Emergency Neurology and Rehabilitation Treatment, N.V. Sklifosovsky Research Institute for Emergency Medicine, Moscow Health Department, 129090 Moscow, Russia; 4 Scobeltsyn Nuclear Physics Research Institute, Lomonosov Moscow State University, 119991 Moscow, Russia

**Keywords:** ischaemic stroke, CT angiography, collateral status, collateral score, scoring, inter-rater reliability

## Abstract

The importance of assessing the collateral status (CS) in patients with ischaemic stroke (IS) has repeatedly been emphasised in clinical guidelines. Various publications offer qualitative or semiquantitative scales with gradations corresponding to the different extents of the collaterals, visualised mostly on the basis of CTA images. However, information on their inter-rater reliability is limited. Therefore, the aim of this study is to investigate the inter-rater reliability of the scales for collateral assessment. CTA images of 158 patients in the acute period of IS were used in the study. The assessment of CS was performed by two experts using three methodologies: the modified Tan scale, the Miteff scale, and the Rosenthal scale. Cohen’s kappa, weighted kappa and Krippendorff’s alpha were used as reliability measures. For the modified Tan scale and the Miteff and Rosenthal scales, the weighted kappa values were 0.72, 0.49 and 0.59, respectively. Although the best measure of consistency was found for the modified Tan scale, no statistically significant differences were revealed among the scales. The impact of the CS on the degree of neurological deficit at discharge was shown for the modified Tan and Rosenthal scales. In conclusion, the analysis showed a moderate inter-rater reliability of the three scales, but was not able to distinguish the best one among them.

## 1. Introduction

Ischaemic stroke (IS) affects over 7.6 million people annually [1] and dominates in the structure of mortality and disability in the population [1,2,3]. Even though mortality rates have decreased by 32% over the last decade [2], up to 78% of survivors have some physical or cognitive deficiency and desperately need extensive rehabilitation [4,5,6]. Reperfusion therapy for IS is aimed at the restoration of the blood flow to the ischaemic brain tissue, and includes two main techniques: intravenous thrombolysis (EVT) with recombinant tissue plasminogen activator and mechanical thrombectomy (MT) [7]. Timely reperfusion therapy can significantly improve the patient’s chances of a complete or partial recovery, but the efficiency of treatment depends not only on the time passed before intervention (onset-to-reperfusion time), but also on the condition of the collateral blood flow that supports the viability of ischaemic areas [8,9,10,11]. The opening of collateral vessels and anastomoses between cerebral arterial vascular territories after thrombosis is one of the pathophysiological stages of IS [12,13].

Digital subtraction angiography (DSA) is considered the gold standard in determining collateral status (CS) [12,14]. However, DSA’s disadvantages, which include invasiveness (it is performed only in patients already selected for reperfusion) and expensiveness, and the fact that it allows imaging only in the territory of the contrasted artery, make the use of other radiological techniques reasonable [15]. Computed tomography angiography (CTA), which is quite common in routine practice and is not time consuming, has demonstrated good concordance with DSA [16,17]. CTA allows high-resolution visualisation of the entire cerebral vasculature and allows precise localisation of stenosis or occlusion. Some studies have found that CTA-assessed CS is even more informative in predicting outcome than assessment performed by means of DSA [18,19].

To date, about two dozen methodologies have been proposed to assess the CS based on CTA images [20,21,22,23,24,25,26,27,28,29,30,31,32]. The simplest scales use only two grades to describe collaterals (poor and good [21,25]). Others use a score from 0 to 10 [28] or up to 20 points [29], taking into consideration the presence of vessels in different brain areas, such as areas corresponding to ASPECTS (Alberta Stroke Program Early CT Score). For visual assessment, authors offer different viewing modes, such as source images (SI), maximum-intensity projection (MPI), multi-planner reformation (MPR) or three-dimensional visualisation. Additionally, some classifications involve not just the characterisation of vessels in the IS area, but their comparison with the intact side [22,27,28,29].

The importance of assessing CS has been repeatedly emphasised in clinical guidelines [33,34,35,36,37], as its main practical relevance is in supporting proper patient selection for reperfusion. Nevertheless, none of the scales has yet been universally accepted. The main reason for this is their low reliability and a very limited number of studies devoted to a comparative analysis of different scales. To date, only a few studies have assessed the inter-rater reliability of existing scales [38,39,40]. Most often, these assessments have been performed by the authors of the scales themselves [20,25,30,32], or in the context of a small number of reviews [38,39,40]. In some studies, the choice of methods for assessing reliability, including the use of intraclass correlation coefficients (ICC) designed to deal with quantitative variables with normal distribution [41], in the task of assessing qualitative scales [29,38,42], is questionable.

The relation of collateral capacity to treatment efficacy and clinical presentation is of great interest in terms of the practical relevance of the scales. To assess it, the authors have most often used the information on the degree of functional independence graded on the modified Rankin Scale (mRs) 3 months after the IS [19,20,39,43]. In reperfusion interventions, favourable outcomes (mRs 0–2) have been found to be significantly more frequent in cases with good collaterals [20,43]. However, it has recently been shown that the NIHSS (National Institutes of Health Stroke Scale) [44] is equally informative as a target outcome variable, with the most pronounced dynamics in the degree of neurological deficit observed after reperfusion interventions and during hospitalisation. To date, there are few works in the literature reflecting the impact of the CS on the clinical performance assessed using the NIHSS upon discharge.

Therefore, the aim of this work was to study the inter-rater reliability of CS scales in IS patients with the use of CTA and analyse their relation to treatment efficacy.

## 2. Materials and Methods

### 2.1. Description of the Sample

This retrospective study used CTA DICOM-images of 158 consecutive patients admitted between 2018 and 2021 to the N.V. Sklifosovsky Research Institute of Emergency Medicine in the acute period of IS. The median age was 72 (IQR 63–81) years, and 46.8% of patients were males. The inclusion criteria were as follows: (1) age of 18 years or older; (2) diagnosis of middle cerebral artery (MCA) ischaemic stroke confirmed with follow-up MRI study (2–3 days after the admission); (3) admission within 6 h after symptoms onset; (4) CTA performed on admission. The exclusion criteria were: (1) poor-quality CTA images and presence of artifacts; (2) absence of CTA images on admission and MR images in dynamics in the hospital PACS (picture archiving and communication system); (3) lacunar pathogenetic variant of IS according to Trial of Org 10,172 in Acute Stroke Treatment (TOAST) classification; (4) lesion not only in MCA territory, but also in the zones of anterior or posterior cerebral artery; (5) bilateral IS.

The degree of neurological deficit was assessed by neurologists with the NIHSS immediately after admission. The standard treatment protocol also included NIHSS score assessment at the following time points: just before reperfusion, every 15 min during EVT, every 30–60 min throughout the first day in the intensive care unit, and then daily. In the current research, we used the information on the NIHSS score at discharge to assess the association of CS with the treatment efficacy. In the case of death due to the underlying disease, a score of 42 was used as the final NIHSS score. In cases of death due to complications (e.g., pulmonary embolism), the data from the last neurological examination before the acute deterioration were recorded.

Non-contrast CT conducted right after the initial neurologic examination and assessed by radiologists showed the absence of visible signs of ischaemia in 82.3% of cases. In 94 cases (59.5%), CTA revealed the presence of MCA occlusion, in 7 cases (4.4%) there was only ICA occlusion. On the follow-up MRI, all patients had confirmed areas of ischaemia in the MCA vascular territory, with an average lesion volume of 111 (IQR 58–164) cm^3^.

Reperfusion therapy was performed in 48.1% of patients: 26 had thrombolysis (EVT) alone, 28 had MT (thromboextraction/thromboaspiration) and 22 had both. The decision on treatment tactics was taken by a team of neurologists and endovascular surgeons in accordance with the National Guidelines [37]. In the case of a positive decision on reperfusion therapy, intravenous thrombolysis was performed by neurologists and resuscitators, and mechanical thrombectomy was performed by endovascular surgeons. For the reperfusion group, the median time from symptoms’ onset to CTA was 95 (IQR 62–143) minutes. EVT was performed right after CT and CTA scanning (directly in the CT room); MT required approximately 20 additional minutes for the transfer to the operating room.

The clinical characteristics of the sample are shown in Table 1 (parameters of statistical tests are available in Table A1 of the Appendix A).

The study was approved by the biomedical ethics committee of the State Budget Institution “N.V. Sklifosovsky Research Institute for Emergency Medicine of the Moscow Health Department” (protocol # 8–22 of 19 July 2022).

### 2.2. Assessment of Collateral Status with CTA Images

The research was performed on a 64-slice Aquilion CXL CT scanner (Toshiba Med. Inc., Tokyo, Japan) with a slice thickness of 0.5 mm. During the first stage, a standard non-contrast brain scan was performed to exclude haemorrhagic components. This was followed by a CTA scan of the brachiocephalic arteries with the aortic arch captured. Data were acquired with 64 × 0.5 mm collimation, and tube voltage and current strength were 120 kVp and 100 mAs, respectively. In all cases, a non-ionic contrast agent with an iodine concentration of at least 350 mg/mL was used that was administered intravenously using an automatic bolus injector at a rate of 4 mL per second, in a volume of 80 mL. An automatic aortic arch bolus tracking system was used to initiate the scan at the right time.

**Figure 1:**
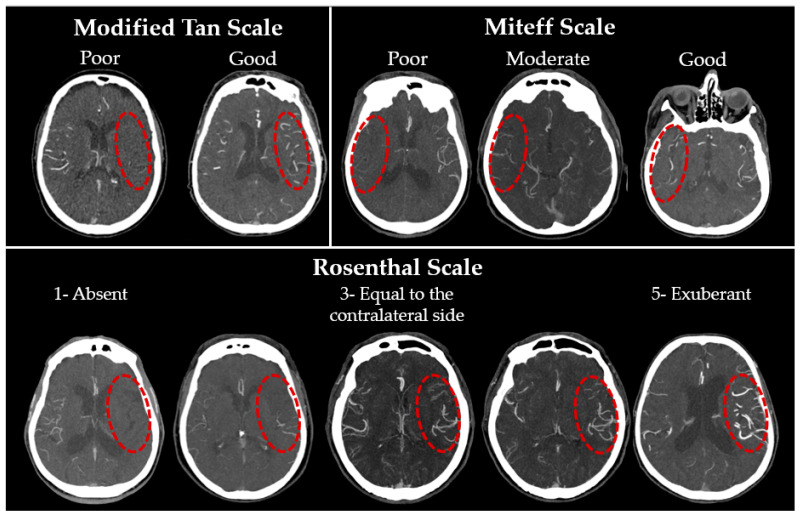
CT angiograms showing examples of collateral status grades for the modified Tan scale, the Miteff scale, and the Rosenthal scale. The areas indicated with a red dashed line indicate the side of the stroke.

The state of the collateral blood flow was assessed using three scales:

(1) The modified Tan scale [25] is based on the binary classification proposed in 2002 by Shramm et al. [21]. This classification is both the simplest and, according to the published works, one of the most reliable [38,39], probably due to the minimal number of grades. A ‘good’ grade is given if collaterals are seen on more than 50% of the MCA basin.
(2) The Miteff scale [20]: the authors propose characterising CS on the basis of the maximum-intensity projection (MIP) according to the following three grades: poor collaterals—only distal superficial branches of the MCA are visible; moderate collaterals—in addition to superficial arteries, the branches of the MCA in the Sylvian sulcus are also visualised; good collaterals—MCA is visualised immediately after the occlusion site. Previously, this system also demonstrated a high inter-rater reliability [39].
(3) The Rosenthal scale [27]: unlike the previous two, this classification is based on the comparison of the affected side with the intact one and includes five gradations: (1) absent vessels; (2) vessels are less than on the contralateral normal side; (3) vessels are represented to the same extent as on the intact side; (4) vessels are greater than on the contralateral normal side; (5) exuberant vessels on the affected side. The authors originally proposed using only CTA-SI, but as MIP and MPR are now firmly integrated into any workstation routine, they were also assessed. Examples of gradations for each scale are presented in Figure 1.

Thus, the modified Tan scale and the Rosenthal scale can both be used for cases with or without visible occlusions, while the Miteff scale is intended only for cases with visible occlusions. The choice of these scales was motivated by the low number of grades, as using them seems not to be so time consuming as that for more detailed scales. Additionally, the modified Tan scale and the Miteff scale were selected because of their relatively high prevalence in the literature and the availability of previously published articles claiming their high reliability, while the Rosenthal scale offered more explicit definitions of grades.

**Figure A1:**
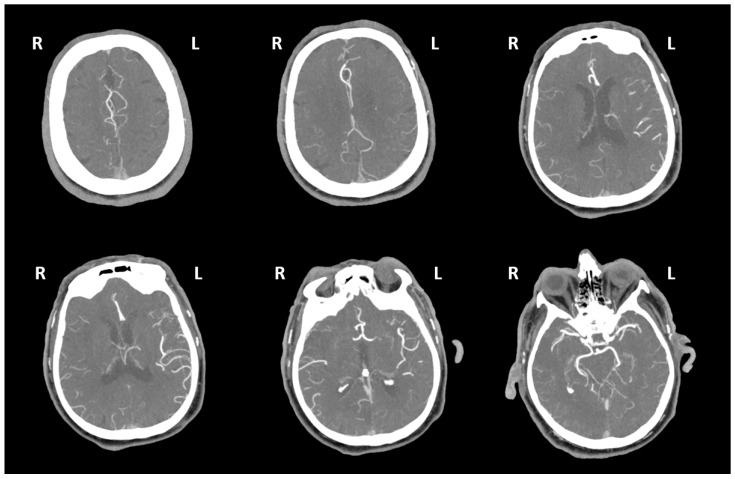
An example of radiologists’ disagreement on the score obtained using the modified Tan scale. Male, 80 years old, with the occlusion of M3 segment of the right MCA. Both experts characterised collateral status as “Good, MCA is visualised immediately after the occlusion site” on the Miteff scale and as “Less than the contralateral normal side” on the Rosenthal scale, but disagreed on the modified Tan scale score.

**Figure A2:**
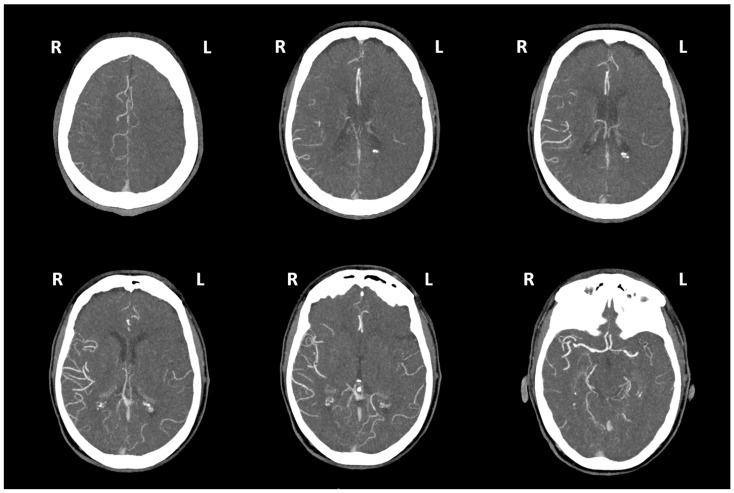
An example of radiologists’ disagreement on the score obtained using the Miteff scale. Female, 76 years old, with the occlusion of M1 segment of the left MCA. Both experts characterised the collateral status as “Poor” on the modified Tan scale and as “Less than the contralateral normal side” on the Rosenthal scale. Nevertheless, one of the radiologists assessed collateral status as “Moderate. Vessels can be seen at the Sylvian fissure”, and the other as “Poor. Only distal superficial branches of the MCA are visible”.

**Figure A3:**
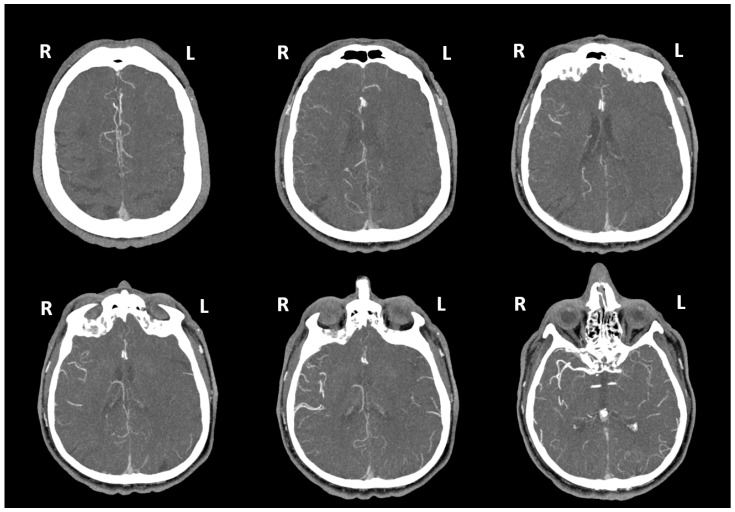
An example of radiologists’ disagreement on the score obtained using the Rosenthal scale. Male, 69 years old, with occlusion of M1 segment of the left MCA. Both experts characterised collateral status as “Poor” on the modified Tan scale and as “Poor. Only distal superficial branches of the MCA are visible” on the Miteff scale. On the Rosenthal scale, there were two opinions: “Less than the contralateral normal side” and “Absent vessels”.

Two radiologists (M.R. and V.S., with 17 and 7 years of experience, respectively) performed the estimation of CS after attending a 1-h training session on the aforementioned scores. The assessment was performed independently and retrospectively, with no additional details on the clinical picture or the results of follow-up radiological studies. The only information provided to the radiologists was the side of the lesion. MIP and MPR were constructed on a Gamma-Multivox workstation. CTA series of three clinical cases, as examples of radiologists’ disagreement, are presented in Appendix B Figure A1, Figure A2 and Figure A3. In the subsequent analysis of the association of CS with the severity of neurological impairment, the scales were binarised, with cases where collaterals were considered poor by at least one expert being combined with those where the experts agreed in their opinion on poor collaterals.

### 2.3. Methods for Assessing Inter-Rater Reliability

There are several methods for assessing the inter-rater reliability of qualitative scales. The most common method is the Cohen’s kappa calculation [45], which is designed to evaluate the consistency of the conclusions of two experts. The interpretation of kappa values is ambiguous, as kappa is sensitive to the balance of groups (the highest values are observed for balanced samples) and to the number of gradations (the more gradations, the lower the kappa) [46]. One of the most common interpretations is the Fleiss version [47], according to which: (1) κ < 0.4 is accepted as poor reliability; (2) 0.4 ≤ κ < 0.75 indicates fair to good reliability; (3) κ ≥ 0.75 indicates excellent reliability.

When a scale has more than two gradations, this methodology does not consider the degree of disagreement between rates. For such cases, it is recommended that a weighted kappa be used [48], the calculation of which involves a system of ‘penalties’ for disagreements (the greater the disagreement between opinions, the greater the ‘penalty’).

Another measure of reliability is Krippendorff’s alpha [49]. This is advantageous when applied to any number of raters, for both quantitative and qualitative data, and is also able to deal with omissions in the data.

### 2.4. Statistical Processing of the Results

Quantitative variables are described in this paper using the median and interquartile range (IQR) because of the small number of cases in most comparison groups and the absence of a normal distribution revealed with the Shapiro–Wilk test. The values of qualitative characteristics are given as absolute and relative frequencies. The Mann–Whitney U test was used to compare quantitative variables, including the NIHSS scores between different groups of CS. A comparison of qualitative characteristics was performed with the Pearson’s chi-square test and Fisher’s exact test. For statistical hypothesis testing, a significance threshold of *p*_o_ = 0.05 was used. A Holm-corrected value of the threshold was also calculated (*p*_o_ = 0.017) in order to address the multiple comparison problem [50].

Cohen’s kappa and weighted kappa were calculated by means of the statistical software package of MedCalc (online version) [51]. The Krippendorffsalpha library was used to assess Krippendorff’s alpha. A point estimate and a 95% confidence interval (95% CI) were calculated for each consistency measure.

The results are visualised using box-and-whiskers plots in which the lower and upper bounds of the box correspond to the first and third quartiles, the line in the middle of the box indicates the median, and the lower and upper whiskers indicate the minimum and maximum values. Outliers are indicated with coloured dots (for observation 1.5 times the interquartile range less than the first quartile or greater than the third quartile) and asterisks (for observation 3 times the interquartile range less than the first quartile or greater than the third quartile).

The statistical analysis was performed in the R Studio environment (version 3.6.1).

## 3. Results

The distribution of the radiologists’ assessments for each of the three scales is given in Table 2, Table 3 and Table 4.

This distribution shows that the sample as a whole was not balanced: there were far fewer cases with poor collaterals. For the Rosenthal scale, the rarest variant was the presence of collaterals greater than on the intact side (Table 4). Additionally, there were no cases with exuberant collaterals on the ischaemic side (grade 5).

The distribution of cases on the Miteff scale for patients with MCA occlusion was relatively even.

The calculation of agreement rates for the three scales (Table 5) showed that the highest coefficient values were observed when using the modified Tan scale.

The resulting weighted kappa values correspond to a moderate degree of inter-rater consistency. However, due to the overlapping confidence intervals, no statistically significant differences can be established with certainty.

**Figure 2:**
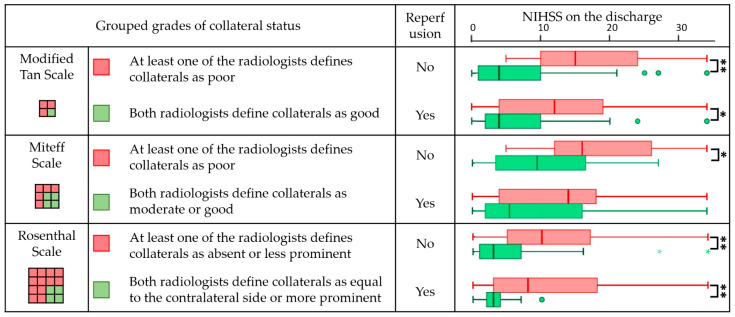
Comparison of patients with poor and good collaterals by NIHSS score on discharge. The symbols * and ** indicate statistically significant differences at *p* < 0.05 and *p* < 0.017 (Holm-corrected value), respectively. Coloured dots and asterisks mark outliers.

The following grouping of the obtained assessments was performed to estimate the influence of CS on the degree of neurological deficit, while taking into account the differences in the examiners’ opinions. For the modified Tan scale and the Miteff scale, both the series where the radiologists’ opinions were unanimous and the series for which only one of the examiners determined the status to be poor were included in the group of cases with poor collaterals. According to the Rosenthal scale, the cases in which at least one examiner rated “no vessels” and “vessels less pronounced than on the intact side” were united in a single group. All other cases were assigned to the group with good CS. Thus, all scales were binarised: only two gradations were identified in each, with conventionally good and poor collaterals, and each case was assigned to one of the gradations. Patients in these groups were compared on the basis of their degree of neurological deficit at discharge with regard to the type of treatment (Figure 2).

The distribution of patients and the descriptive statistics for NIHSS scores on discharge are presented in Table 6 (the parameters of statistical tests are presented in Table A2 in the Appendix A).

The evaluation of the impact of CS on the degree of neurological deficit on discharge showed statistically significant differences for the modified Tan scale and Rosenthal scale: regardless of whether reperfusion interventions were performed or not, the NIHSS score at discharge was significantly lower in the group of patients with good collaterals (marked green in the box diagrams). For the Miteff scale, a significant difference was registered only for the patients under conservative treatment (*p* = 0.031), possibly due to the small number of observations in the groups.

## 4. Discussion

The state of CS is one of the key factors in the course of IS. Unfortunately, the definition of this term remains one of the vaguest. It is important to note that when analysing the CT and MRI images, we did not refer to the visualisation of leptomeningeal anastomoses (also known as pial collateral vessels), which are less than 1 mm in diameter, but rather larger-calibre vessels that were retrogradely filled from them [13,15].

Most often, in the context of collateral blood flow assessment, CT and MR perfusion are mentioned, as they allow the visualisation of the core and penumbra zones, with the latter being more prominent due to good collaterals [11]. However, not every hospital is capable of performing these studies, making the analysis of CTA images more relevant. Previous works on the application of scales in the assessment of CS by CTA indicate the presence of the relationship of obtained scores with the outcome of IS and the effectiveness of reperfusion interventions [43,52,53]. However, there is limited information on the inter-rater agreement of the scales as a measure of their reliability [20,28,39,40,43].

In this study, we investigated the three scales with the lowest numbers of grades; two of them have demonstrated high reliability on the metrics used in several publications. For example, in the study by Weiss et al. [40], the weighted kappa values for the modified Tan scale and the Miteff scale were 0.86 and 0.81, respectively (95% CI not presented), while Yeo et al. [39] reported even higher reliability values in a CTA analysis of 200 patients: the Cohen kappa values for the modified Tan scale and the Miteff scale were 0.93 (95% CI 0.91–0.95) and 0.91 (95% CI 0.86–0.93), respectively. The authors of the Miteff scale themselves reported similar results (Cohen’s kappa = 0.93) on data from 92 patients [20], which may be due to the homogeneous nature of the sample: only patients with occlusion in the M1 segment were included.

The inter-rater reliability assessed in the current study was characterised by far more modest results. The weighted Cohen’s kappa values for the modified Tan scale and the Miteff scale were 0.72 (95% CI 0.59, 0.84) and 0.56 (95% CI 0.41, 0.71), respectively, indicating that both point estimates and confidence intervals were significantly lower than the reported values. At the same time, the Multicentre Randomised Clinical Trial of Endovascular Treatment of Acute Ischaemic Stroke (MR CLEAN) study of 493 patients was also known to have a moderate inter-rater consistency, with a weighted kappa of only 0.49 for the modified Tan scale [43].

One possible reason for such a wide distribution of consistency measurements is the somewhat variable manner in which the scales are used: some articles simply mention the imaging of the arteries in the ischaemic area when describing the collateral assessment method [20,25], while others explicitly state the need for a comparison with the intact side for the same scales [40]. The viewing parameters (SI alone [21,25], MPR alone [25,29], MIP alone [19,20,25] or MPR along with MIP [40]) may also have some influence. As the generation of MPR and MIP has become a routine practice for radiologists, in our work, we used both. At the same time, there are no clear recommendations regarding the thickness of MIP reconstruction for the assessment of the intracranial vascular bed. In our work, MIP images with a thickness of at least 10 mm were the best, in the opinion of the examining radiologists.

For the Rosenthal five-grade scale, inter-rater agreement had not been assessed before, either by the authors of the scale or by other research teams, so the current work is the first study of the reliability of this scale [27]. In terms of the results obtained, no significant difference in reliability was detected compared with the other two. In the literature, classification is widely performed based on the five Rosenthal grades: according to the 10-point M.B. Maas scale, the Sylvian sulcus vessels and leptomeningeal arteries [28] are to be separately characterised using the five-point scale; however, the assessment of the inter-rater reliability of this scale was beyond the scope of this study.

The comparison with most other scales, including more detailed ones, does not suggest the use of the latter to be preferable over these scales. For example, in a study by Yang et al., in which the inter-rater reliability was estimated on the basis of data from 100 CTA series, the original four-grade Tan scale and the authors’ proposed expanded six-grade version of this scale produced weighted kappa values of 0.65 (95% CI 0.42–0.88) and 0.7 (95% CI 0.59–0.81), respectively [30]. Unfortunately, it was impossible to correlate our results with those presented in some publications in which the same collateral status scales were used due to incorrect statistical analyses and the uninformative nature of the indicators provided by the authors [38].

It is important to note that in the majority of studies, the authors, who had initially used more detailed gradations, enlarged the groups and reduced the number of gradations to two in the subsequent analysis of the association with the degree of functional independence (mRs), as well as in attempts to construct prognostic rules [19,52,54].

The use of the NIHSS score at the end of hospitalisation as an output variable is a distinctive feature of the current study: previously published studies focused on mRs 3 months after IS in order to assess the impact of CS on clinical outcome [19,39,55]. The possibility of using the NIHSS as a surrogate endpoint instead of mRs has already been demonstrated in the literature [44]. We believe that, despite the differences in the purpose of these scales (NIHSS for describing neurological deficits and mRs for describing functional independence), this substitution has several justifications. Firstly, it is during the intrahospital period rather than during rehabilitation that the most pronounced progression in the patient’s status is observed; secondly, the need to follow patients up for 3 months often results in the loss of some cases, and therefore smaller sample sizes. In addition, the use of mRs is time consuming and costly. There are few studies analysing the relationship between CS and NIHSS assessment one day after symptom onset (early neurological improvement) [32,56]. Since our study included patients on conservative therapy, who do not commonly show dramatic improvements one day after onset, we assessed the degree of neurological deficit at the time of discharge.

Another particularity of our work was the inclusion of both patients with visible occlusions for whom endovascular interventions had been performed and patients who, for various reasons, were receiving conservative treatment alone. Most works are devoted to the assessment of the association of collaterals with the clinical outcome in patients after reperfusion [19,39,55,56]; however, in cases where interventions are contraindicated or the occlusion site cannot be visualised, the outcome of IS will be almost entirely determined by collateral reserves. Our data showed that the presence of good collaterals assessed on the basis of the modified Tan and Rosenthal scales was a significant predictor of less severe neurological symptoms in this group of patients.

Overall, our results are fully consistent with those of other authors based on the data from the mRs: the presence of good collaterals causes a less severe clinical picture and a greater efficacy of reperfusion interventions.

None of the publications we analysed used Krippendorff’s alpha to assess consistency, and the lack of the original cross-tabulations prevented us from calculating it by ourselves. Since there remains some ambiguity in the assessment of CS, additional multicentre studies involving multiple radiologists and employing both operator-dependent scales and more quantitative methods of collateral assessment, which are clearly the trend of recent research, are necessary.

One of the most promising solutions to the problem of the subjectivity of CS assessment is the development of algorithms for the automatic assessment of cerebral vessels [57,58]. So far, only a few clinically validated software products have been presented (e.g., e-STROKE by Brainomix Ltd., StrokeViewer by Nico-lab), and the results of their implementation into routine practice indicate a significant potential of this direction of CTA image analysis [10,30,42]. Moreover, similar software can be applied for IS in vertebrobasilar system, for which there are currently different scales from those for CMA IS [59,60,61,62]. In these cases, the use of the Krippendorff’s alpha will allow the results of different scales and different study designs to be compared.

The limitations of this study are as follows. Firstly, it was a relatively small sample, in which the distribution of patients into comparison groups was unbalanced: gradations with poor collaterals, assessed using the modified Tan and Rosenthal scales, were much less frequent. However, judging by the fact that a similar distribution has been observed in many other studies [20,43,52,56,57], it may reflect the situation in the population in general. Nevertheless, small groups and the non-normality of the distribution of variables resulted in nonparametric tests having less statistical power than parametric ones. Secondly, it is obvious that the single-phase CTA is sensitive to temporal information and does not allow the visualisation of vessel filling in later phases. There is evidence in the literature suggesting two- and three-phase CTA as being more informative [32,63,64,65], but in practice these studies are not as widespread as single-phase CTA. Finally, the study design was single-centred and non-randomised, which may result in biases.

Additionally, it should be mentioned that the study design did not take into account the structure of the circle of Willis, which acts as the primary collateral system, as well as the presence of extra-intracranial anastomoses [9]. Their influence on the stroke outcome was studied in previously published articles [23,66,67], but due to the considerable variety in their anatomies (including combinations of aplasia/hypoplasia of anterior and posterior communicating arteries, as well as of A1 and P1 segments), an integrated approach and representative samples are necessary for future research.

We believe that the findings presented in the current work can help in planning further studies with higher evidential value, in particular through preliminary power analysis and meta-analysis. These steps will make it possible to come closer to solving the problem of performing reliable collateral assessment and proper patient selection for reperfusion therapy, which is especially important for those who have been admitted outside the therapeutic window or who have an unknown time of symptom onset (wake-up strokes).

## 5. Conclusions

The analysis of the CS assessment by two radiologists using CTA images demonstrated a moderate degree of inter-rater reliability. The highest rates of consistency were observed for the modified Tan scale, but there were no statistically significant differences among the scales. Significant differences were revealed when assessing the relationship between the CS and the dynamics of neurological deficit: patients with good collaterals on the modified Tan and Rosenthal scales received lower values for their NIHSS score. This pattern was observed in both the group of patients after reperfusion and in the group undergoing conservative treatment alone. The objectification of the CS assessment based on CTA images remains an unsolved and urgent task that requires further research.

## Figures and Tables

**Table 1:** Characteristics of the study population (comparison of groups was performed using the Mann–Whitney U test for quantitative variables, the Pearson’s chi-squared test and the Fisher’s exact test for qualitative variables. The symbol * denotes statistically significant results).

Characteristics	Overall (n = 158)	No Reperfusion (n = 82)	Reperfusion (n = 76)	*p*-Value
Age, years, median (IQR)	72 (63–81)	72 (62–82)	71 (63–80)	0.578
Gender, male, n (%)	74 (46.8%)	37 (45.1%)	37 (48.7%)	0.750
NIHSS score on the admission, median (IQR)	11 (6–18)	8 (4–18)	14 (9–18)	0.003 *
Time to CTA, minutes, median (IQR)	117 (84–222)	180 (101–306)	95 (62–143)	≪0.001 *
ASPECTS score on the admission:				
No visible signs of ischaemia (ASPECTS = 10), n (%)	130 (82.3%)	64 (78.0%)	66 (86.8%)	0.211
Visible signs of ischaemia (ASPECTS < 10), n (%)	28 (17.7%)	18 (22.0%)	10 (13.2%)	
ASPECTS score, median (IQR)	7 (6–8)	8 (7–8)	6 (6–7)	0.038 *
IS subtypes according to the TOAST classification:				
Large-artery atherosclerosis, n (%)	28 (17.7%)	16 (19.5%)	12 (15.8%)	
Cardioembolic, n (%)	63 (39.9%)	25 (30.5%)	38 (50.0%)	0.041 *
Stroke of undetermined aetiology, n (%)	63 (39.9%)	38 (46.3%)	25 (32.9%)	
Stroke of other determined aetiology, n (%)	4 (2.5%)	3 (3.7%)	1 (1.3%)	
State of major cerebral arteries:				
No visible occlusions, n (%)	57 (36.1%)	43 (52.4%)	14 (18.4%)	
Occlusion of the MCA, n (%)	94 (59.5%)	35 (42.7%)	59 (77.6%)	≪0.001 *
Occlusion only of the ICA, n (%)	7 (4.4%)	4 (4.9%)	3 (3.9%)	
NIHSS score on the discharge, median (IQR)	6 (2–14)	5 (2–13)	7 (2–16)	0.638
Death, n (%)	48 (30.4%)	25 (30.5%)	23 (30.3%)	1.000

NIHSS, National Institutes of Health Stroke Scale; CTA, computed tomography angiography, ASPECTS, The Alberta Stroke Program Early CT Score; IS, ischaemic stroke; TOAST, Trial of Org 10,172 in Acute Stroke Treatment; MCA, middle cerebral artery; ICA, internal carotid artery.

**Table 2:** Distribution of radiologists’ opinions on the CS score for the modified Tan scale.

Radiologist #1	Radiologist #2	
	Poor Collaterals	Good Collaterals
Poor collaterals, n	32	10
Good collaterals, n	7	109

**Table 3:** Distribution of radiologists’ opinions on CS score for the Miteff scale.

Radiologist #1	Radiologist #2		
	Poor Collaterals	Moderate Collaterals	Good Collaterals
Poor collaterals, n	16	2	-
Moderate collaterals, n	9	21	6
Good collaterals, n	5	14	21

**Table 4:** Distribution of radiologists’ opinions on CS score for the Rosenthal scale.

Radiologist #1	Radiologist #2			
	1—Absent	2—Less than the Contralateral Normal Side	3—Equal to the Contralateral Normal Side	4—Greater than the Contralateral Normal Side
1—Absent, n	9	11	-	-
2—Less than the contralateral normal side, n	3	41	8	1
3—Equal to the contralateral normal side, n	-	21	57	2
4—Greater than the contralateral normal side, n	-	-	2	3

**Table 5:** Inter-rater reliability scores for the three scales assessing collateral status. The 95% confidence interval is shown in parentheses.

Consistency Measure	Modified Tan Scale	Miteff Scale	Rosenthal Scale
Cohen’s kappa (unweighted)	0.72 (95% CI 0.59, 0.84)	0.42 (95% CI 0.28, 0.57)	0.51 (95% CI 0.40, 0.62)
Kappa with linear weighting	0.72 (95% CI 0.59, 0.84)	0.49 (95% CI 0.35, 0.63)	0.59 (95% CI 0.49, 0.69)
Krippendorff’s alpha	0.72 (95% CI 0.59, 0.83)	0.55 (95% CI 0.38, 0.70)	0.69 (95% CI 0.60, 0.76)

**Table 6:** Distribution of NIHSS scores on discharge in patients, grouped by CS grade. The symbols * and ** indicate statistically significant differences at *p* < 0.05 and *p* < 0.017 (Holm-corrected value), respectively.

	Scale Name	No Reperfusion Therapy			Reperfusion Therapy		
		Poor Collaterals	Good Collaterals	M-W Test	Poor Collaterals	Good Collaterals	M-W Test
NIHSS score on discharge, median (IQR)	Modified Tan scale	15 (10–26) n = 15	4 (1–10) n = 67	≪0.001 **	12 (4–19) n = 34	4 (2–10) n = 42	0.019 *
	Miteff Scale	16 (10–26) n = 11	10 (3–17) n = 24	0.031 *	14 (4–19) n = 21	6 (2–16) n = 38	0.129
	Rosenthal Scale	10 (5–18) n = 37	3 (1–8) n = 45	0.001 **	8 (3–18) n = 57	3 (2–4) n = 19	0.003 **

NIHSS, National Institutes of Health Stroke Scale.

## Data Availability

Data available upon reasonable request.

## Appendix A

**Table A1:** Characteristics of the study population with parameters of statistical tests. The symbol * indicates statistically significant differences.

Characteristics	Overall (n = 158)	No Reperfusion (n = 82)	Reperfusion (n = 76)	*p*-Value
Age, years, median (IQR)	72 (63–81) *p* (S-W) ≪ 0.001 W = 0.965	72 (62–82) *p* (S-W) = 0.004 W = 0.953	71 (63–80) *p* (S-W) = 0.084 W = 0.971	*p* (M-W) = 0.578 Z = −0.557
Gender, male, n (%)	74 (46.8%)	37 (45.1%)	37 (48.7%)	*p* (Fisher’s exact) = 0.750
NIHSS score on the admission, median (IQR)	11 (6–18) *p* (S-W) ≪ 0.001 W = 0.949	8 (4–18) *p* (S-W) ≪ 0.001 W = 0.894	14 (9–18) *p* (S-W) = 0.047 W = 0.967	*p* (M-W) = 0.003 * Z = 2.941
Time to CTA, minutes, median (IQR)	117 (84–222) *p* (S-W) ≪ 0.001 W = 0.322	180 (101–306) *p* (S-W) ≪ 0.001 W = 0.424	95 (62–143) *p* (S-W) ≪ 0.001 W = 0.288	*p* (M-W)≪0.001 * Z = −4.659
ASPECTS score on the admission:				
No visible signs of ischaemia (ASPECTS = 10), n (%)	130 (82.3%)	64 (78.0%)	33 (86.8%)	*p* (Fisher’s exact) = 0.211
Visible signs of ischaemia (ASPECTS < 10), n (%)	28 (17.7%)	18 (22.0%)	10 (13.2%)	
ASPECTS score, median (IQR)	7 (6–8) *p* (S-W) = 0.006 W = 0.889	8 (7–8) *p* (S-W) = 0.002 W = 0.801	6 (6–7) *p* (S-W) = 0.286 W = 0.911	*p* (M-W) = 0.038 * Z = −2.073
IS subtypes according to the TOAST classification:				
Large-artery atherosclerosis, n (%)	28 (17.7%)	16 (19.5%)	12 (15.8%)	*p* (Pearson’s χ^2^) =0.041 * χ^2^ = 6.394
Cardioembolic, n (%)	63 (39.9%)	25 (30.5%)	38 (50.0%)	
Stroke of undetermined aetiology, n (%)	63 (39.9%)	38 (46.3%)	25 (32.9%)	
Stroke of other determined aetiology, n (%)	4 (2.5%)	3 (3.7%)	1 (1.3%)	
State of major cerebral arteries:				
No visible occlusions, n (%)	57 (36.1%)	43 (52.4%)	14 (18.4%)	*p* (Fisher’s exact) ≪ 0.001 *
Occlusion of the MCA, n (%)	94 (59.5%)	35 (42.7%)	59 (77.6%)	
Occlusion only of the ICA, n (%)	7 (4.4%)	4 (4.9%)	3 (3.9%)	
NIHSS score on the discharge, median (IQR)	6 (2–14) *p* (S-W) ≪ 0.001 W = 0.848	5 (2–13) *p* (S-W) ≪ 0.001 W = 0.836	7 (2–16) *p* (S-W) ≪ 0.001 W = 0.856	*p* (M-W) = 0.638 Z = 0.468
Death, n (%)	48 (30.4%)	25 (30.5%)	23 (30.3%)	*p* (Fisher’s exact) = 1.000

NIHSS, National Institutes of Health Stroke Scale; CTA, computed tomography angiography, ASPECTS, The Alberta Stroke Program Early CT Score; IS, ischaemic stroke; TOAST, Trial of Org 10,172 in Acute Stroke Treatment; MCA, middle cerebral artery; ICA, internal carotid artery.

**Table A2:** NIHSS score on discharge in united CS grades. The symbols * and ** indicate statistically significant differences between poor and good collaterals groups calculated with the Mann–Whitney U test, *p* < 0.05 and *p* < 0.017 (Holm-corrected value), respectively. The Shapiro–Wilk test results demonstrate a check for the normality of distributions.

Scale Name	No Reperfusion Therapy			Reperfusion Therapy		
	Poor Collaterals	Good Collaterals	M-W Test	Poor Collaterals	Good Collaterals	M-W Test
Modified Tan scale	15 (10–26) n = 15 *p* (S-W) = 0.200 W = 0.921	4 (1–10) n = 67 *p* (S-W) ≪ 0.001 W = 0.793	*p* ≪ 0.001 ** Z = 3.845	12 (4–19) n = 34 *p* (S-W) = 0.021 W = 0.924	4 (2–10) n = 42 *p* (S-W) ≪ 0.001 W = 0.763	0.019 * Z = 2.347
Miteff Scale	16 (10–26) n = 11 *p* (S-W) = 0.599 W = 0.958	10 (3–17) n = 24 *p* (S-W) = 0.165 W = 0.926	*p* = 0.031 * Z = 2.152	14 (4–19) n = 21 *p* (S-W) = 0.016 W = 0.924	6 (2–16) n = 38 *p* (S-W) = 0.001 W = 0.825	0.129 Z = 1.516
Rosenthal Scale	10 (5–18) n = 37 *p* (S-W) = 0.484 W = 0.961	3 (1–8) n = 45 *p* (S-W) ≪ 0.001 W = 0.749	*p* = 0.001 ** Z = 3.473	8 (3–18) n = 57 *p* (S-W) = 0.005 W = 0.915	3 (2–4) n = 19 *p* (S-W) ≪ 0.001 W = 0.811	0.003 ** Z = 3.020

## References

[1] Feigin V.L., Brainin M., Norrving B., Martins S., Sacco R.L., Hacke W., Fisher M., Pandian J., Lindsay P. (2022). World Stroke Organization (WSO): Global stroke fact sheet 2022. Int. J. Stroke.

[2] Feigin V.L., Stark B.A., Johnson C.O., Roth G.A., Bisignano C., Abady G.G., Abbasifard M., Abbasi-Kangevari M., Abd-Allah F., Abedi V. (2021). Global, Regional, and National Burden of Stroke and Its Risk Factors, 1990–2019: A Systematic Analysis for the Global Burden of Disease Study 2019. Lancet Neurol..

[3] Tsao C.W., Aday A.W., Almarzooq Z.I., Alonso A., Beaton A.Z., Bittencourt M.S., Boehme A.K., Buxton A.E., Carson A.P., Commodore-Mensah Y. (2022). Heart Disease and Stroke Statistics—2022 Update: A Report from the American Heart Association. Circulation.

[4] Lv Y., Sun Q., Li J., Zhang W., He Y., Zhou Y. (2021). Disability Status and Its Influencing Factors among Stroke Patients in Northeast China: A 3-Year Follow-up Study. Neuropsychiatr. Dis. Treat..

[5] do Carmo J.F., Morelato R.L., Pinto H.P., de Oliveira E.R.A. (2015). Disability after Stroke: A Systematic Review. Fisioter. Em Mov..

[6] de Almeida Moraes M., Mussi F.C., Muniz L.S., Sampaio E.E.S., de Sena Leitão T., de Souza Teles Santos C.A., de Jesus P.A.P. (2022). Clinical Characterization, Disability, and Mortality in People with Strokes during 90 Days. Rev. Bras. Enferm..

[7] Imran R., Mohamed G.A., Nahab F. (2021). Acute Reperfusion Therapies for Acute Ischemic Stroke. J. Clin. Med..

[8] Malhotra K., Liebeskind D.S. (2020). Collaterals in Ischemic Stroke. Brain Hemorrhages.

[9] Maguida G., Shuaib A. (2023). Collateral Circulation in Ischemic Stroke: An Updated Review. J. Stroke.

[10] Uniken Venema S.M., Wolff L., van den Berg S.A., Reinink H., Luijten S.P.R., Lingsma H.F., Marquering H.A., Boers A.M.M., Bot J., Hammer S. (2022). Time Since Stroke Onset, Quantitative Collateral Score, and Functional Outcome after Endovascular Treatment for Acute Ischemic Stroke. Neurology.

[11] Jung S., Wiest R., Gralla J., McKinley R., Mattle H., Liebeskind D. (2017). Relevance of the Cerebral Collateral Circulation in Ischaemic Stroke: Time is Brain, but Collaterals Set the Pace. Swiss Med. Wkly.

[12] Ravindran A.V., Killingsworth M.C., Bhaskar S. (2020). Cerebral Collaterals in Acute Ischaemia: Implications for Acute Ischaemic Stroke Patients Receiving Reperfusion Therapy. Eur. J. Neurosci..

[13] Kaloss A.M., Theus M.H. (2022). Leptomeningeal Anastomoses: Mechanisms of Pial Collateral Remodeling in Ischemic Stroke. WIREs Mech. Dis..

[14] Shaban S., Huasen B., Haridas A., Killingsworth M., Worthington J., Jabbour P., Bhaskar S.M.M. (2021). Digital Subtraction Angiography in Cerebrovascular Disease: Current Practice and Perspectives on Diagnosis, Acute Treatment and Prognosis. Acta Neurol. Belg..

[15] Raymond S.B., Schaefer P.W. (2017). Imaging Brain Collaterals. Top. Magn. Reson. Imaging.

[16] Lee S.J., Liu B., Rane N., Mitchell P., Dowling R., Yan B. (2021). Correlation between CT Angiography and Digital Subtraction Angiography in Acute Ischemic Strokes. Clin. Neurol. Neurosurg..

[17] Lu W.-Z., Lin H.-A., Hou S.-K., Bai C.-H., Lin S.-F. (2022). Diagnostic Test Accuracy of Pretreatment Collateral Score in Predicting Stroke Outcomes after Intra-Arterial Endovascular Thrombectomy: A Meta-Analysis in DSA and CTA. Eur. Radiol..

[18] Jansen I.E., Berkhemer O.A., Yoo A.J., Vos J.A., Lycklama à Nijeholt G.J., Sprengers M.E.S., van Zwam W.H., Schonewille W.J., Boiten J., van Walderveen M.A.A. (2016). Comparison of CTA- and DSA-Based Collateral Flow Assessment in Patients with Anterior Circulation Stroke. Am. J. Neuroradiol..

[19] Kauw F., Dankbaar J.W., Martin B.W., Ding V.Y., Boothroyd D.B., van Ommen F., de Jong H., Kappelle L.J., Velthuis B.K., Heit J.J. (2020). Collateral Status in Ischemic Stroke: A Comparison of Computed Tomography Angiography, Computed Tomography Perfusion, and Digital Subtraction Angiography. J. Comput. Assist. Tomogr..

[20] Miteff F., Levi C.R., Bateman G.A., Spratt N., McElduff P., Parsons M.W. (2009). The Independent Predictive Utility of Computed Tomography Angiographic Collateral Status in Acute Ischaemic Stroke. Brain.

[21] Schramm P., Schellinger P.D., Fiebach J.B., Heiland S., Jansen O., Knauth M., Hacke W., Sartor K. (2002). Comparison of CT and CT Angiography Source Images with Diffusion-Weighted Imaging in Patients with Acute Stroke within 6 Hours after Onset. Stroke.

[22] Casault C., Al Sultan A.S., Trivedi A., Sohn S.I., Qazi E., Bokyo M., Almekhlafi M., d’Esterre C., Goyal M., Demchuk A.M. (2017). Collateral Scoring on CT Angiogram Must Evaluate Phase and Regional Pattern. Can. J. Neurol. Sci..

[23] Sundaram S., Kannoth S., Thomas B., Sarma P.S., Sylaja P.N. (2016). Collateral Assessment by CT Angiography as a Predictor of Outcome in Symptomatic Cervical Internal Carotid Artery Occlusion. Am. J. Neuroradiol..

[24] Jia B., Liebeskind D.S., Song L., Xu X., Sun X., Liu L., Wang B., Miao Z. (2017). Performance of Computed Tomography Angiography to Determine Anterograde and Collateral Blood Flow Status in Patients with Symptomatic Middle Cerebral Artery Stenosis. Interv. Neuroradiol..

[25] Tan J.C., Dillon W.P., Liu S., Adler F., Smith W.S., Wintermark M. (2007). Systematic Comparison of Perfusion-CT and CT-Angiography in Acute Stroke Patients. Ann. Neurol..

[26] Knauth M., von Kummer R., Jansen O., Hähnel S., Dörfler A., Sartor K. (1997). Potential of CT Angiography in Acute Ischemic Stroke. Am. J. Neuroradiol..

[27] Rosenthal E., Schwamm L.H., Roccatagliata L., Coutts S.B., Demchuk A.M., Schaefer P.W., Gonzalez R.G., Hill M.D., Halpern E.F., Lev M.H. (2008). Role of Recanalization in Acute Stroke Outcome: Rationale for a CT Angiogram-Based “Benefit of Recanalization” Model. Am. J. Neuroradiol..

[28] Maas M.B., Lev M.H., Ay H., Singhal A.B., Greer D.M., Smith W.S., Harris G.J., Halpern E., Kemmling A., Koroshetz W.J. (2009). Collateral Vessels on CT Angiography Predict Outcome in Acute Ischemic Stroke. Stroke.

[29] Menon B.K., Smith E.J., Modi J., Patel S.R., Bhatia R.S., Watson T.F., Hill M.D., Demchuk A.M., Goyal M. (2011). Regional Leptomeningeal Score on CT Angiography Predicts Clinical and Imaging Outcomes in Patients with Acute Anterior Circulation Occlusions. Am. J. Neuroradiol..

[30] Yang W., Soomro J., Jansen H., Venkatesh A., Yoo A.J., Lopes D.K., Beenen M., Emmer B.J., Majoie C.B.L.M., Marquering H.A. (2023). Collateral Capacity Assessment. Clin. Neuroradiol..

[31] Dolotova D.D., Blagosklonova E.R., Ramazanov G.R., Arkhipov I.V., Petrikov S.S., Gavrilov A. (2022). Evaluation of Cerebral Collateral Status Using Computed Tomography Angiography in Ischemic Stroke: Review of Manual and Automated Methods. Nejrohirurgiâ.

[32] Menon B.K., d’Esterre C.D., Qazi E.M., Almekhlafi M., Hahn L., Demchuk A.M., Goyal M. (2015). Multiphase CT Angiography: A New Tool for the Imaging Triage of Patients with Acute Ischemic Stroke. Radiology.

[33] Powers W.J., Rabinstein A.A., Ackerson T., Adeoye O.M., Bambakidis N.C., Becker K., Biller J., Brown M., Demaerschalk B.M., Hoh B. (2019). Guidelines for the Early Management of Patients with Acute Ischemic Stroke: 2019 Update to the 2018 Guidelines for the Early Management of Acute Ischemic Stroke: A Guideline for Healthcare Professionals from the American Heart Association/American Stroke Association. Stroke.

[34] Liu L., Ding J., Leng X., Pu Y., Huang L.-A., Xu A., Wong K.S.L., Wang X., Wang Y. (2018). Guidelines for Evaluation and Management of Cerebral Collateral Circulation in Ischaemic Stroke 2017. Stroke Vasc. Neurol..

[35] Heran M., Lindsay P., Gubitz G., Yu A., Ganesh A., Lund R., Arsenault S., Bickford D., Derbyshire D., Doucette S. (2022). Canadian Stroke Best Practice Recommendations: Acute Stroke Management, 7thEdition Practice Guidelines Update, 2022. Can. J. Neurol. Sci..

[36] Turc G., Bhogal P., Fischer U., Khatri P., Lobotesis K., Mazighi M., Schellinger P.D., Toni D., de Vries J., White P. (2019). European Stroke Organisation (ESO)—European Society for Minimally Invasive Neurological Therapy (ESMINT) Guidelines on Mechanical Thrombectomy in Acute Ischaemic StrokeEndorsed by Stroke Alliance for Europe (SAFE). Eur. Stroke J..

[37] Russian Sociaty of Neurologists (2021). Clinical Guidlines of the Russian Ministry of Healthcare. Ischaemic Stroke and Transient Ischaemic Attack in Adults.

[38] Wolff L., Su J., Van Loon D., van Es A., van Doormaal P.J., Majoie C., van Zwam W., Dippel D., van der Lugt A., van Walsum T. (2022). Inter-Rater Reliability for Assessing Intracranial Collaterals in Patients with Acute Ischemic Stroke: Comparing 29 Raters and an Artificial Intelligence-Based Software. Neuroradiology.

[39] Yeo L.L.L., Paliwal P., Teoh H.L., Seet R.C., Chan B.P., Ting E., Venketasubramanian N., Leow W.K., Wakerley B., Kusama Y. (2014). Assessment of Intracranial Collaterals on CT Angiography in Anterior Circulation Acute Ischemic Stroke. AJNR Am. J. Neuroradiol..

[40] Weiss D.J., Kraus B., Rubbert C., Jander S., Gliem M., Lee J.-I., Haensch C.-A., Turowski B., Caspers J. (2019). Systematic Evaluation of Computed Tomography Angiography Collateral Scores for Estimation of Long-Term Outcome after Mechanical Thrombectomy in Acute Ischaemic Stroke. Neuroradiol. J..

[41] Bobak C.A., Barr P.J., O’Malley A.J. (2018). Estimation of an Inter-Rater Intra-Class Correlation Coefficient That Overcomes Common Assumption Violations in the Assessment of HRealth Measurement Scales. BMC Med. Res. Methodol..

[42] Grunwald I.Q., Kulikovski J., Reith W., Gerry S., Namias R., Politi M., Papanagiotou P., Essig M., Mathur S., Joly O. (2019). Collateral Automation for Triage in Stroke: Evaluating Automated Scoring of Collaterals in Acute Stroke on Computed Tomography Scans. Cerebrovasc. Dis..

[43] Berkhemer O.A., Jansen I.G.H., Beumer D., Fransen P.S.S., van den Berg L.A., Yoo A.J., Lingsma H.F., Sprengers M.E.S., Jenniskens S.F.M., Lycklama À. (2016). Collateral Status on Baseline Computed Tomographic Angiography and Intra-Arterial Treatment Effect in Patients with Proximal Anterior Circulation Stroke. Stroke.

[44] Chalos V., van der Ende N.A.M., Lingsma H.F., Mulder M.J.H.L., Venema E., Dijkland S.A., Berkhemer O.A., Yoo A.J., Broderick J.P., Palesch Y.Y. (2020). National Institutes of Health Stroke Scale. Stroke.

[45] Cohen J. (1960). A Coefficient of Agreement for Nominal Scales. Educ. Psychol. Meas..

[46] Maclure M., Willett W.C. (1987). Misinterpretation and Misuse of the Kappa Statistic. Am. J. Epidemiol..

[47] Fleiss J.L., Levin B., Paik M.C. (2003). Statistical Methods for Rates and Proportions.

[48] Fleiss J.L., Cohen J., Everitt B.S. (1969). Large Sample Standard Errors of Kappa and Weighted Kappa. Psychol. Bull..

[49] Krippendorff K. (2004). Reliability in Content Analysis: Some Common Misconceptions and Recommendations. Hum. Commun. Res..

[50] Holm S. (1979). A Simple Sequentially Rejective Multiple Test Procedure. Scand. J. Stat..

[51] MedCalc Software Ltd. Inter-Rater Agreement. https://www.medcalc.org/calc/kappa.php.

[52] Kim H.S., Lee S.-J., Lee T.-K. (2019). Pretreatment Collateral Status Predicts Malignant Stroke Evolution in Patients Undergoing Endovascular Thrombectomy. J. Neurosonol. Neuroimag..

[53] Leng X., Lan L., Liu L., Leung T.F., Yau H. (2016). Good Collateral Circulation Predicts Favorable Outcomes in Intravenous Thrombolysis: A Systematic Review and Meta-Analysis. Eur. J. Neurol..

[54] Rebchuk A.D., Field T.S., Hill M.D., Goyal M., Demchuk A.M., Holodinsky J.K., Fainardi E., Shankar J., Najm M., Rubiera M. (2022). Determinants of Leptomeningeal Collateral Status Variability in Ischemic Stroke Patients. Can. J. Neurol. Sci.

[55] Alqahtani S., Alnaami I., Alhazzani A., Alahmari F., Wassel Y.I., Elsayed E., Abdrabou A., Bassiouny Mohamed A.-A. (2023). Correlation between Pre-Treatment Collateral Status and Short-Term Functional Outcome in Patients with Mild to Moderate Stroke after Reperfusion Therapy in a Local Primary Stroke Center in the Southwestern Part of Saudi Arabia. Cureus.

[56] Faizy T.D., Mlynash M., Kabiri R., Christensen S., Kuraitis G., Meyer L., Bechstein M., van Horn N., Lansberg M.G., Albers G.W. (2022). Favourable Arterial, Tissue-Level and Venous Collaterals Correlate with Early Neurological Improvement after Successful Thrombectomy Treatment of Acute Ischaemic Stroke. J. Neurol. Neurosurg. Psychiatry.

[57] Su J., Wolff L., van Es A.C.G.M., van Zwam W., Majoie C., Dippel D.W.J., van der Lugt A., Niessen W.J., Van Walsum T. (2020). Automatic Collateral Scoring from 3D CTA Images. IEEE Trans. Med. Imaging.

[58] Kuang H., Wan W., Wang Y., Wang J., Qiu W. (2023). Automated Collateral Scoring on CT Angiography of Patients with Acute Ischemic Stroke Using Hybrid CNN and Transformer Network. Biomedicines.

[59] Filep R.C., Mărginean L., Stoian A., Bajko Z. (2021). Diagnostic and Prognostic Computed Tomography Imaging Markers in Basilar Artery Occlusion (Review). Exp. Ther. Med..

[60] Alemseged F., Shah D.G., Diomedi M., Sallustio F., Bivard A., Sharma G., Mitchell P.J., Dowling R.J., Bush S., Yan B. (2017). The Basilar Artery on Computed Tomography Angiography Prognostic Score for Basilar Artery Occlusion. Stroke.

[61] van der Hoeven E.J., McVerry F., Vos J.A., Algra A., Puetz V., Kappelle L.J., Schonewille W.J. (2016). Collateral Flow Predicts Outcome after Basilar Artery Occlusion: The Posterior Circulation Collateral Score. Int. J. Stroke.

[62] Da Ros V., Meschini A., Gandini R., Del Giudice C., Garaci F., Stanzione P., Rizzato B., Diomedi M., Simonetti G., Floris R. (2016). Proposal for a Vascular Computed Tomography-Based Grading System in Posterior Circulation Stroke: A Single-Center Experience. J. Stroke Cerebrovasc. Dis..

[63] Shin N.-Y., Kim K., Park M., Kim Y.D., Kim D.J., Ahn S.J., Heo J.H., Lee S.-K. (2014). Dual-Phase CT Collateral Score: A Predictor of Clinical Outcome in Patients with Acute Ischemic Stroke. PLoS ONE.

[64] Wang Z., Xie J., Tang T.-Y., Zeng C.-H., Zhang Y., Zhao Z., Zhao D.-L., Geng L.-Y., Deng G., Zhang Z.-J. (2020). Collateral Status at Single-Phase and Multiphase CT Angiography versus CT Perfusion for Outcome Prediction in Anterior Circulation Acute Ischemic Stroke. Radiology.

[65] Seker F., Pereira-Zimmermann B., Pfaff J., Purrucker J.C., Gumbinger C., Schönenberger S., Bendszus M., Möhlenbruch M.A. (2019). Collateral Scores in Acute Ischemic Stroke. Clin. Neuroradiol..

[66] Zhou H., Sun J., Ji X., Lin J., Tang S., Zeng J., Fan Y. (2016). Correlation between the Integrity of the Circle of Willis and the Severity of Initial Noncardiac Cerebral Infarction and Clinical Prognosis. Medicine.

[67] Oumer M., Alemayehu M., Muche A. (2021). Association between Circle of Willis and Ischemic Stroke: A Systematic Review and Meta-Analysis. BMC Neurosci..

